# Case report: Emerging species in post-traumatic endophthalmitis: *Acinetobacter johnsonii*

**DOI:** 10.3389/fmed.2024.1406277

**Published:** 2024-08-13

**Authors:** Jiezhong Hu, Chunling Huang, Jingyi Li, Caixia Fang, Jiali Li, Songfu Feng

**Affiliations:** ^1^Department of Ophthalmology, Houjie Hospital, Dongguan, China; ^2^Department of Ophthalmology, Zhujiang Hospital, Southern Medical University, Guangzhou, China

**Keywords:** endophthalmitis, *Acinetobacter johnsonii*, pathogen identification, open globe injury, antibiotic susceptibility

## Abstract

*Acinetobacter johnsonii* is an uncommon cause of endophthalmitis. This case report describes a 40-year-old male admitted with pain, redness, and vision loss in his right eye after an open globe injury by a steel fragment. Clinical assessment confirmed post-traumatic endophthalmitis with an intraocular foreign body. The patient underwent a vitreous biopsy, lensectomy, vitrectomy, and intravitreal antibiotics, followed by laser photocoagulation and foreign body extraction via the pars plana. Acinetobacter johnsonii was isolated from the vitreous culture. A combination of vancomycin, levofloxacin and ceftazidime was administered, leading to reduced infection and inflammation. Postoperatively at one month, the patients’ best-corrected visual acuity had improved to 20/63. The anterior segment exhibited no inflammation, the vitreous cavity was clear, and the retina with hemorrhage and laser treatment remained stable. The one-year follow-up confirmed the continued stability of the ocular condition. *Acinetobacter johnsonii*, a rare cause of endophthalmitis often linked to trauma or surgery, should be recognized as a possible pathogen in post-traumatic endophthalmitis cases, meriting clinical consideration.

## 1 Introduction

Ocular trauma, particularly those associated with intraocular foreign bodies (IOFBs), is a serious ocular disease that may lead to disastrous consequences, including endophthalmitis, phthisis bulbi, and irreversible vision loss (1). The epidemiology of ocular trauma is influenced by various factors such as the patient’s age, region, socioeconomic status, and sporting activity (2). Post-traumatic endophthalmitis is a serious ocular disease that leads to significant visual impairment following open globe injuries, with incidence rates between 4 and 16% (3). The delayed treatment of traumatic endophthalmitis may result in irreversible damage to visual function, and in severe cases, it can lead to blindness or even exophthalmia. The treatment of posterior segment IOFBs is challenging due to the complex clinical presentation and the necessity to achieve multiple goals during the treatment process, including ocular integrity preservation, foreign body removal, and functional recovery. Currently, pre-pars plana vitrectomy (PPV) is considered as the preferred method for managing conditions that impact the posterior segment of the eye, including the presence of retained IOFBs (4). The most prevalent microorganism in traumatic endophthalmitis is Staphylococcus epidermidis, and it is common for multiple microorganisms to be involved (5, 6). Early intervention, pathogen virulence, and antibiotic resistance patterns affect the development and prognosis of post-traumatic endophthalmitis (7). While endophthalmitis due to common pathogens like staphylococci and *Pseudomonas aeruginosa* is well-documented, *Acinetobacter johnsonii*, an opportunistic pathogen from aquatic and dermal sources with a low human isolation rate of 1.7 to 2.0% (8), has not been previously reported in this context. We report a rare case of post-traumatic endophthalmitis caused by *Acinetobacter johnsonii*, highlighting its unique pathogenic profile and clinical management.

## 2 Case description

**FIGURE 1:**
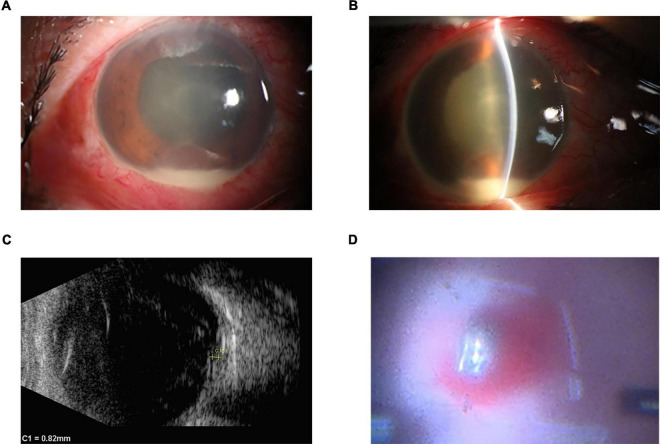
Initial clinical appearance of right eye. **(A,B)** Slit lamp examination showed ocular hyperemia, 2.0 mm hypopyon, fibrinous membrane, and severe cellular reaction (grade 4 cells) in the anterior chamber, which obscured further visualization of the lens and fundus. **(C)** The images were acquired using a 10-MHz probe (Aviso, Quantel Medical), showing a foreign body accompanied by dense opacities located anterior to the retina on B-scan ultrasonography. **(D)** The surgical microscope showed the foreign body was in front of the retina with retinal hemorrhage.

A 40-year-old man was urgently transported to a local hospital three days ago following an open globe injury by a steel fragment from an automobile machine. Initially, he did not complain any symptoms of erythema, pain, or visual impairment in his right eye and was treated with erythromycin eye ointment at the local hospital. However, on the following day, he developed with symptoms of pain, redness and a decreased vision in the affected eye, which gradually worsened. Consequently, the patient returned to the local hospital for a follow-up visit and the computerized tomography scan showed an intraocular foreign body in his right eye. The local hospital recommended referring the patient to our hospital due to the concern for potential post-traumatic endophthalmitis. The patient was subsequently transferred to our hospital on the third day post-injury. His best-corrected visual acuity in the affected right eye was reduced to counting fingers at 10 cm while the left eye maintained normal acuity at 20/20. The intraocular pressure was 25 mmHg in the right eye and 14mmHg in the left, as measured by non-contact tonometer. Upon slit lamp examination, the right eye showed ocular hyperemia and a self-sealing entry site in the nasal conjunctiva and sclera. There was 2.0 mm hypopyon, fibrinous membrane, and severe cellular reaction (grade 4 cells) in the anterior chamber, which obscured further visualization of the lens and fundus (Figure 1). The left eye’s anterior segment and fundus were without any abnormalities. The patient underwent a thorough examination of the vitreous body and retina using a 10MHz linear array ultrasound probe. Utilizing ocular B-mode ultrasonography (AVISO, Quantel Medical), both transverse scan and longitudinal scans revealed the presence of a foreign body accompanied by dense opacities located anterior to the retina (Figure 1). Collectively, these clinical observations confirmed a diagnosis of post-traumatic endophthalmitis in the right eye, complicated by an intraocular foreign body.

Eight hours after diagnosis, the patient underwent a standard undiluted vitreous biopsy, lensectomy, vitrectomy, intravitreal injection of antibiotics, laser photocoagulation, and removal of the foreign body (Figure 1). The surgeon utilized a 23G trocar forscleral punctures at the 2, 7, and 10 o’clock position, precisely placed 3.5 mm from the corneal limbus. A 3mm conjunctival and scleral laceration was observed at the 3 o’clock nasal position, 4mm from the corneal limbus. The conjunctiva and sclera were incised to probe the wound, and no foreign bodies were detected. After suturing the laceration with 8-0 silk sutures, the optical fiber, perfusion head, and vitreous cutter were sequentially placed. An intraoperative finding of lenticular opalescence compromised the visual field, necessitating a lensectomy. A 2mm incision was made at the 3 o’clock position at the corneal limbus, complemented by a 1mm auxiliary incision at the 2 o’clock position. The vitreous cutter was introduced to clear the anterior chamber of fibrinous exudates and purulent material during vitrectomy, extensive subretinal hemorrhage in the retinal posterior pole and profuse villous excrescences on the anterior retina were observed. A copper metal fragment measuring approximately 2*3 mm was observed in the posterior pole of the retina. The scleral puncture site at the 10 o’clock position was dilated to 3mm to facilitate the extraction of the foreign body with forceps. The surgeon applied retinal laser photocoagulation and administered intravitreal antibiotics (vancomycin 1.0mg/0.1ml and ceftazidime 2.25mg/0.1ml). The procedure concluded with the meticulous closure using 8-0 silk sutures.

**FIGURE 2:**
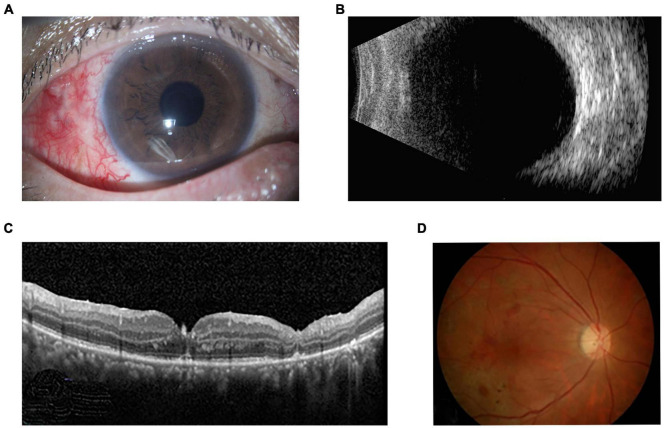
One-month postoperative appearance of right eye. **(A)** Slit lamp examination showed the there was no inflammation in the anterior segment. **(B)** Ultrasonography revealed a clear vitreous cavity with an attached retina. **(C)** OCT and **(D)** fundus photograph showed retinal hemorrhage, laser spots and attached retina with retinal atrophy.

The patient was administered intravenous antibiotics, including 2.0 g ceftazidime twice daily for 5 days and 0.5 g levofloxacin once daily for 10 days. Before obtaining the susceptibility results, the initial selection of these antibiotics was informed by several considerations. Firstly, both antibiotics showed a broad-spectrum activity against a range of Gram-negative bacteria, which aligns with our clinical findings, Secondly, we considered the pharmacokinetic and pharmacodynamic profiles of the antibiotics. Ceftazidime and levofloxacin have favorable ocular penetration and tissue distribution, which are crucial for achieving effective intraocular concentrations. Additionally, we took into account the potential for adverse effects and drug interactions. Both selected antibiotics have a well-established safety profile, which is an important consideration in clinical practice. Lastly, the availability and cost-effectiveness of these antibiotics in our healthcare setting were also considered. Ceftazidime and levofloxacin are widely accessible and affordable, making them practical choices for the treatment regimen. Vitreous culture revealed the organism was *Acinetobacter johnsonii*. The organism was sensitive *in vitro* to ceftazidime, imipenem, meropenem, ampicillin/sulbactam, cefoperazone/sulbactam, ciprofloxacin, levofloxacin, amikacin, gentamicin, trimethoprim/sulfamethoxazole, doxycycline, tobramycin and tigecycline. And the drug susceptibility results demonstrated that levofloxacin was the most sensitive agents against Acinetobacter. Therefore, the intravenous injection of cephalosporin was discontinued after the drug susceptibility results were obtained, and the only levofloxacin was administered intravenously. The patient also used eye drops including hormone, 0.5% compound tropicamide, levofloxacin, ceftazidine and vancomycin. Postoperatively at one month, the patients’ best-corrected visual acuity had improved to 20/63. The anterior segment exhibited no inflammation, the vitreous cavity was clear, and the retina with hemorrhage laser treatment remained stable (Figure 2). The one-year follow-up confirmed the continued stability of the ocular condition.

## 3 Discussion

*Acinetobacter johnsonii* is a rare but emerging cause of endophthalmitis. Classified within the Moraxellaceae family, this non-motile, oxidase-negative, Gram-negative bacterium (9, 10), has been isolated from diverse environments, including water bodies, human skin, and animals (11). It causes a range of clinical infections in humans, including catheter-related bloodstream infection and peritonitis associated with peritoneal dialysis (10). As for post-traumatic endophthalmitis, the most common strains of Acinetobacter are *A. baumannii*, *A. calcoaceticus* and *A. lwofi* (12). To our best knowledge, this is the first report of post-traumatic endophthalmitis induced by *Acinetobacter johnsonii* in the English peer review literature. This case highlighted the association of *Acinetobacter johnsonii* with post-traumatic endophthalmitis.

The clinical manifestations of *Acinetobacter johnsonii* endophthalmitis parallel those of endophthalmitis infected by other microbial agents (13, 14). In infectious endophthalmitis, pathogens such as Staphylococcus aureus, Streptococcus species, and Bacillus subtilis are noted for their potent toxicity, swift progression, and severe tissue damage, often linked to the presence of intraocular foreign bodies and portending a poor prognosis (5, 6). The typical symptoms of pain, ocular redness, and vision loss, as exhibited in our case, are common among most patients with infectious endophthalmitis (15). *Acinetobacter johnsonii* is characterized by a short incubation period and marked infectiousness in cases of post-traumatic endophthalmitis. The prompt onset in this instance implies a highly virulent nature of the bacterium. It is customary for infectious endophthalmitis to swiftly evolve into panophthalmitis within the initial 24 h (5). A striking feature of *Acinetobacter johnsonii* endophthalmitis was that inflammation mainly manifested in the anterior segment rather than the posterior segment. Therefore, we should carefully distinguish between traumatic acute inflammatory response and true endophthalmitis. Firstly, Endophthalmitis is more likely to occur in the presence of risk factors such as retained intraocular foreign bodies and perforating ocular trauma (16). Traumatic acute inflammatory response usually occurs immediately after the injury, while true endophthalmitis may have a more delayed onset (17). Secondly, in endophthalmitis, there is often a more pronounced cellular reaction with increased cells and flare in the anterior chamber, fibrinoid exudation, and in severe cases, hypopyon (pus in the anterior chamber). While both conditions can present with vitreous clouding, it tends to be more severe and extensive in endophthalmitis, potentially involving the entire vitreous cavity. The retina and choroid in endophthalmitis may show signs of infection such as retinitis, choroiditis, or abscess formation, which are not features of a non-infectious inflammatory response (15). Thirdly, Endophthalmitis tends to have a progressive and worsening clinical course if left untreated, whereas an acute inflammatory response may stabilize or improve with appropriate management (16).

In certain clinical situations, it is common to start broad-spectrum antibiotics before bacterial culture is available, especially when there is a high risk of serious infection or when the condition is rapidly worsening (15). The rise of antimicrobial resistance poses an increasing danger to the attainment of favorable treatment results in ocular infections, placing patients in jeopardy of vision loss or even the loss of their entire eye. According to a study that focused on post-traumatic endophthalmitis along with antimicrobial resistance data, it was found that Gram-positive cocci exhibited resistance to various antibiotics. The resistance rates were as follows: chloramphenicol (4%), aminoglycosides (amikacin 44%, gentamicin 26%), fluoroquinolones (gatifloxacin 10%, ciprofloxacin 26%, moxifloxacin 14%, ofloxacin 19%), cephalosporins (cefazolin 10%, ceftazidime 42%), and vancomycin 2%. Similarly, Gram-positive bacilli also demonstrated resistance to all tested antimicrobials, with a higher resistance rate observed for ceftazidime (84%). In the case of P. aeruginosa isolates, resistance rates were found to be 69% for chloramphenicol, 25% for gentamicin, and 31% for ciprofloxacin (18–20). In addition to multidrug resistance, antibiotics exhibit numerous side effects. Therefore, in the field of ophthalmology, antiseptics have emerged as a relevant treatment option due to their broad spectrum of non-selective mechanisms of action (21). The use of silicone oil tamponade may enhance the antimicrobial activity of intravenous antibiotics, aiding in more effective infection control. It also supports the anatomical structure, leading to a more stable and improved final visual outcome (22). Some researchers have suggested the application of preservative-free 0.6% povidone iodine eye drops for patients undergoing intravitreal injections, as this can achieve a better suppressive effect on conjunctival bacteria (23). However, the selection of antibiotic must be customized for each patient. The issue of antimicrobial resistance among Acinetobacter species is a serious concern (24–26). While the drug sensitivity profile of *Acinetobacter baumannii* has been relatively well-documented, there is a scarcity of research on the susceptibility patterns of *Acinetobacter johnsonii* (11). This case adds to the literature on post-traumatic endophthalmitis caused by *Acinetobacter johnsonii* and offers insights into its drug sensitivity patterns, which is valuable for guiding clinical treatment decisions.

In conclusion, this case report highlights the emerging role of *Acinetobacter johnsonii* as a potential pathogen of post-traumatic endophthalmitis. *Acinetobacter johnsonii* is highly virulent with a short incubation period in post-traumatic endophthalmitis and clinical manifestations mainly affect the anterior segment of the eye. Clinicians should be aware of this potential pathogen of post-traumatic endophthalmitis. To facilitate early diagnosis and optimal treatment, it is important to master microbiological details and clinical findings of *Acinetobacter johnsonii* endophthalmitis.

## Data availability statement

The original contributions presented in the study are included in the article/supplementary material, further inquiries can be directed to the corresponding authors.

## Ethics statement

Written informed consent was obtained from the individual(s) for the publication of any potentially identifiable images or data included in this article.

## Author contributions

JH: Conceptualization, Methodology, Writing−original draft. CH: Conceptualization, Methodology, Writing−original draft. JYL: Writing−original draft. CF: Writing−original draft. SF: Funding acquisition, Methodology, Project administration, Writing−review and editing. JLL: Funding acquisition, Methodology, Project administration, Writing−review and editing.
